# Intense bone fluorescence reveals hidden patterns in pumpkin toadlets

**DOI:** 10.1038/s41598-019-41959-8

**Published:** 2019-03-29

**Authors:** Sandra Goutte, Matthew J. Mason, Marta M. Antoniazzi, Carlos Jared, Didier Merle, Lilian Cazes, Luís Felipe Toledo, Hanane el-Hafci, Stéphane Pallu, Hugues Portier, Stefan Schramm, Pierre Gueriau, Mathieu Thoury

**Affiliations:** 1 Laboratório de História Natural de Anfíbios Brasileiros (LaHNAB), Departamento de Biologia Animal, Instituto de Biologia, Universidade Estadual de Campinas, Campinas, São Paulo 13083-862 Brazil; 2 New York University Abu Dhabi, Saadiyat Island, Abu Dhabi, United Arab Emirates; 3 Department of Physiology, Development & Neuroscience, University of Cambridge, Downing Street, Cambridge, CB2 3EG United Kingdom; 4 Laboratory of Cell Biology, Instituto Butantan, São Paulo, 05503-900 Brazil; 5 Sorbonne Universités, CR2P (CNRS, MNHN, UPMC), Muséum national d’Histoire naturelle. CP38, 8, rue Buffon, 75005, Paris, France; 6 B2OA UMR 7052, Université Paris Diderot, Sorbonne Paris Cité, CNRS, F-75010 Paris, France; 7 B2OA UMR 7052, Ecole Nationale Vétérinaire d’Alfort, Université Paris-Est, F- 94700 Maisons-Alfort, France; 8 COST, Université d’Orléans, 45100 Orléans, France; 9 IPANEMA, CNRS, ministère de la Culture; UVSQ, USR 3461, Université Paris-Saclay, F-91192 Gif-sur-Yvette, France; 10 Institute of Earth Sciences, University of Lausanne, Géopolis, CH-1015 Lausanne, Switzerland

## Abstract

The phenomenon of fluorescence can be used by animals to change effective colouration or patterning, potentially to serve functions including intra- and interspecific signalling. Initially believed to be restricted to marine animals, fluorescent colours are now being described in an increasing number of terrestrial species. Here, we describe unique, highly fluorescent patterns in two species of pumpkin toadlets (*Brachycephalus ephippium* and *B*. *pitanga*). We establish that the origin of the fluorescence lies in the dermal bone of the head and back, visible through a particularly thin skin. By comparing them to those of the closely related species *Ischnocnema parva*, we demonstrate that pumpkin toadlets’ bones are exceptionally fluorescent. We characterize the luminescence properties of the toadlets’ bones and discuss the potential function of fluorescent patterns in natural lighting conditions.

## Introduction

Luminescence induced by absorption of photons, i.e. photoluminescence, is the process by which an electron from an atom, molecule, or crystal in an excited state undergoes a transition to a lower energy state, e.g. the ground state, and in doing so, emits a photon. Fluorescence is a particular case of photoluminescence occurring when photons in the short wavelength range, such as ultra-violet (UV), are absorbed by a molecule bearing a fluorophore and re-emitted at longer wavelengths. In fluorescence, the electronic energy transition responsible for the emission does not change in electron spin, in contrast with the electronic energy transition occurring in phosphorescence. Among terrestrial species, fluorescence has recently been described in birds^1^, spiders^2^, frogs^3^ and chameleons^4^. Depending on the visual range and sensitivity of the observer, fluorescence may increase overall conspicuousness of an individual against the background^5^, serve as camouflage^6^ or provide additional information, if the fluorescent patterns differ from the colour patterns visible under non-UV light (but see^7^ for an example of fluorescent patterns not contributing to visual signals produced by toxic butterflies). Fluorescent patches in parrots and spiders have been shown to play a role in sexual communication^1,2^.

Most compounds generating fluorescence have been found in external tissues, such as the cuticle of some invertebrates (spiders^2^, shrimps^8^, scorpions^9^), the feathers of some parrots^1^, and the skin of fishes^5,10,11^, a marine turtle^12^ and certain frogs^3,13^. Externally-visible fluorescent bone has been recently described in chameleons^4^, but never before in any other vertebrates. Here, we describe fluorescent patterns created by ossified tissues, visible through the skin of two species of pumpkin toadlets (*Brachycephalus ephippium* and *B*. *pitanga*; Brachycephalidae). We characterize these fluorescent structures using a combination of histological fluorescence and X-ray imaging techniques, compare them with those of close relatives of similar size, *Brachycephalus hermogenesi* and *Ischnocnema parva*, and discuss their potential functions.

## Results

### A unique fluorescence in pumpkin toadlets

**Figure 1:**
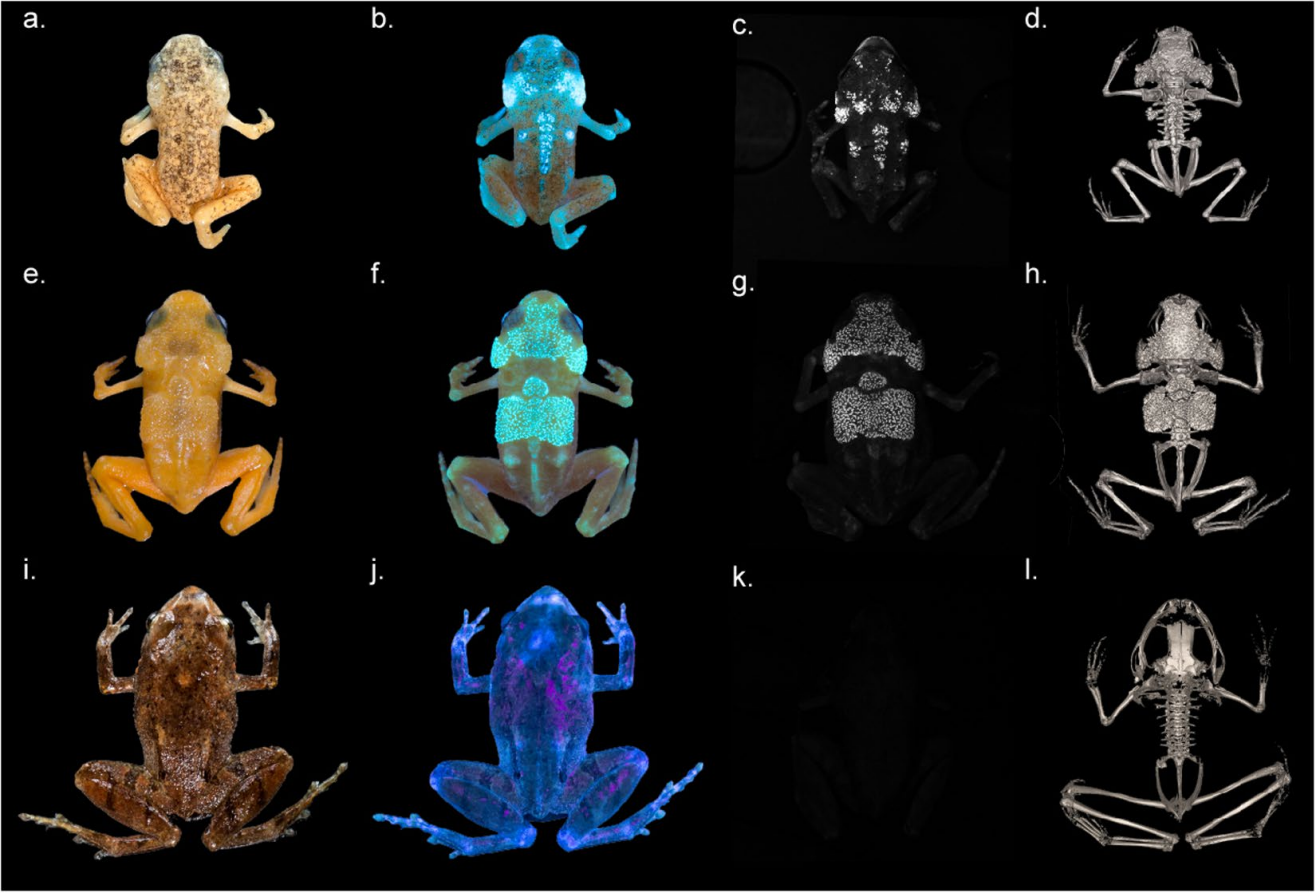
Fluorescence in pumpkin toadlets. Ethanol-preserved specimens of *Brachycephalus pitanga* (**a**–**c**), *B*. *ephippium* (**e**–**g**) and *Ischnocnema parva* (k), and live *Ischnocnema parva* (**i**,**j**) photographed in natural light (**a**,**e**,**i**) and showing fluorescence under UV illumination using two Fluotest Forte UV (λ_excitation_ centred around 365 nm; **b**,**f**,**j**) and a laboratory UV light source (λ_excitation_ = 365 nm) and an emission filter centred around 472 nm and 30 nm wide, thereby eliminating reflectance of all visible light (**c**,**g**,**k**). Note that the absence of fluorescence in *I*. *parva* results in a completely dark image (**k**). Computerized micro-tomography (µCT) reconstructions (**c**,**h**,**l**) show the correspondence between fluorescent patterns and bone structure in *B*. *pitanga* (**d**), *B*. *ephippium* (**h**) and *I*. *parva* (**l**). Photographs taken by L.C. and S.G. (**a**,**b**,**e**,**f**,**i**,**j**) and P.G., M.T. and S.G. (**c**,**g**,**k**).

**Figure 2:**
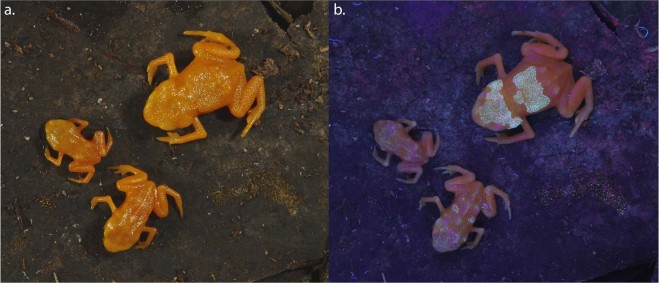
Development of fluorescence in *Brachycephalus ephippium*. Three live individuals of *B*. *ephippium* share the same skin colour (**a:** natural lighting) but present different levels of fluorescence (**b;** UV illumination using two SANKYO lamps λ_excitation_ = 315–400 nm, no filter), corresponding to the extent of dermal ossification. The smallest individual (juvenile) does not present any fluorescent pattern; the larger juvenile presents several fluorescent points on its back and head; the adult presents the complete pattern, with more obvious fluorescence of the head and back. Photographs taken by S.G. and C.J.

Pumpkin toadlets are a radiation of small anurans inhabiting the Atlantic forest of Brazil^14^. All species are diurnal, and many species are toxic and brightly coloured (yellow, orange or red), such as *Brachycephalus ephippium* and *B*. *pitanga*^14^. Under illumination within the UV-A spectral range (λ = 365–390 nm), these two species show prominent emission patterns in the visible range, appearing as bright, whitish patches composed of small spots on the otherwise orange skin. These patterns are not distinguishable to the human eye under regular lighting (Fig. 1a–c,e–g; Video S1). Three-dimensional modelling of the skeleton through micro-computed tomography shows that the fluorescent patterns correspond to the dermal ossifications on the heads and backs of the toadlets, which form plates made of tens of bony tubercles (Fig. 1d,h). Fluorescent patterns are present in both sexes but are absent in the youngest individuals, before the dermal ossification develops (Fig. 2). Ten similarly-sized *Brachycephalus ephippium* individuals were dissected to check sexual organs and vocal slits, indicative of maturity. Sexually mature individuals (N = 7) presented a more developed dorsal plate than similarly-sized sub-adults (N = 3; Fig. S1). Fluorescence in individuals with fully-developed dorsal plates appeared yellowish, whereas fluorescence in sub-adults and juveniles appeared blueish. This is probably indicative of a slightly thicker epidermal layer over the ossified dermis in adults. No specimen of *Brachycephalus pitanga* was dissected (all individuals were mature individuals, collected while calling or carrying eggs visible through the abdominal wall), but we can assume a similar, late-stage development of the dermal ossification.

### Anatomical origin of pumpkin toadlets’ fluorescence

**Figure 3:**
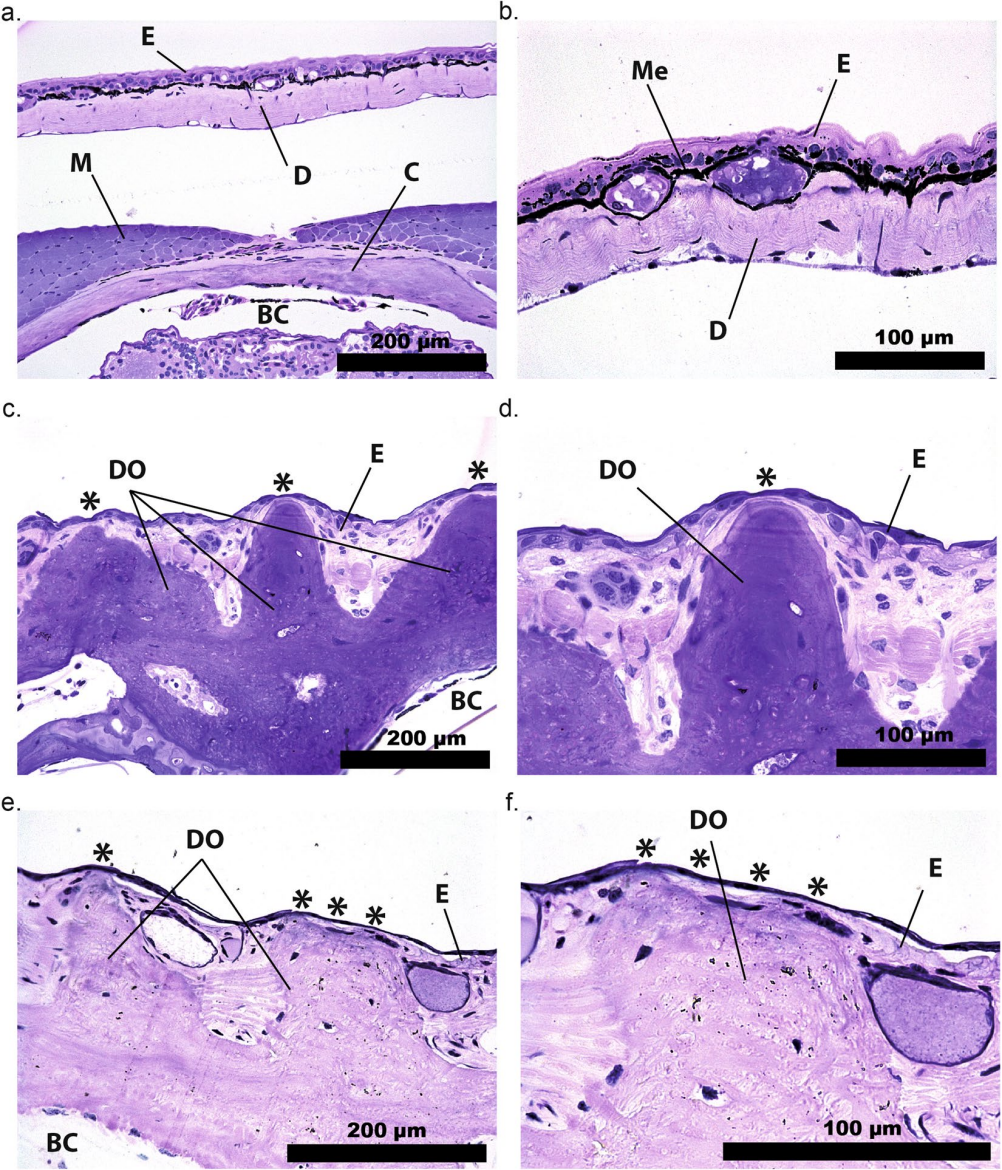
Dermal ossification in pumpkin toadlets. Photomicrographs of transverse histological sections of heads of *Brachycephalus hermogenesi* (**a**,**b**), *B*. *ephippium* (**c**,**d**) and *B*. *pitanga* (**e**,**f**). BC: brain cavity, C: cranial bone, D: dermis, DO: dermal ossification, E: epidermis, M: muscle, Me: melanophore. Asterisks indicate points where the fluorescent ossified tissue is visible through the thin epidermis in live specimens. *B*. *hermogenesi* lacks dermal ossification and a layer of melanophores is present, in contrast to *B*. *ephippium* and *B*. *pitanga*.

Histological sections of the fluorescent regions of the head and back reveal that the ossified dermal plates lie directly below a very thin layer of epidermis, in both *Brachycephalus ephippium* and *B*. *pitanga* (Fig. 3). The epidermis above the dermal ossification is as thin as 7 µm in places where the fluorescence would be externally visible, and 40–140 µm between tubercles. Both *Brachycephalus* species lack melanophores, in contrast to the congeneric *B*. *hermogenesi* (Fig. 3). Melanophores are dark-pigmented cells lying between the epidermis and the dermis of amphibians which block the passage of light, especially short wavelengths such as UV^15–17^. The thinness of the epidermal layer over the bony tubercles and the lack of melanophores allow the bone fluorescence to be externally visible in pumpkin toadlets.

**Figure 4:**
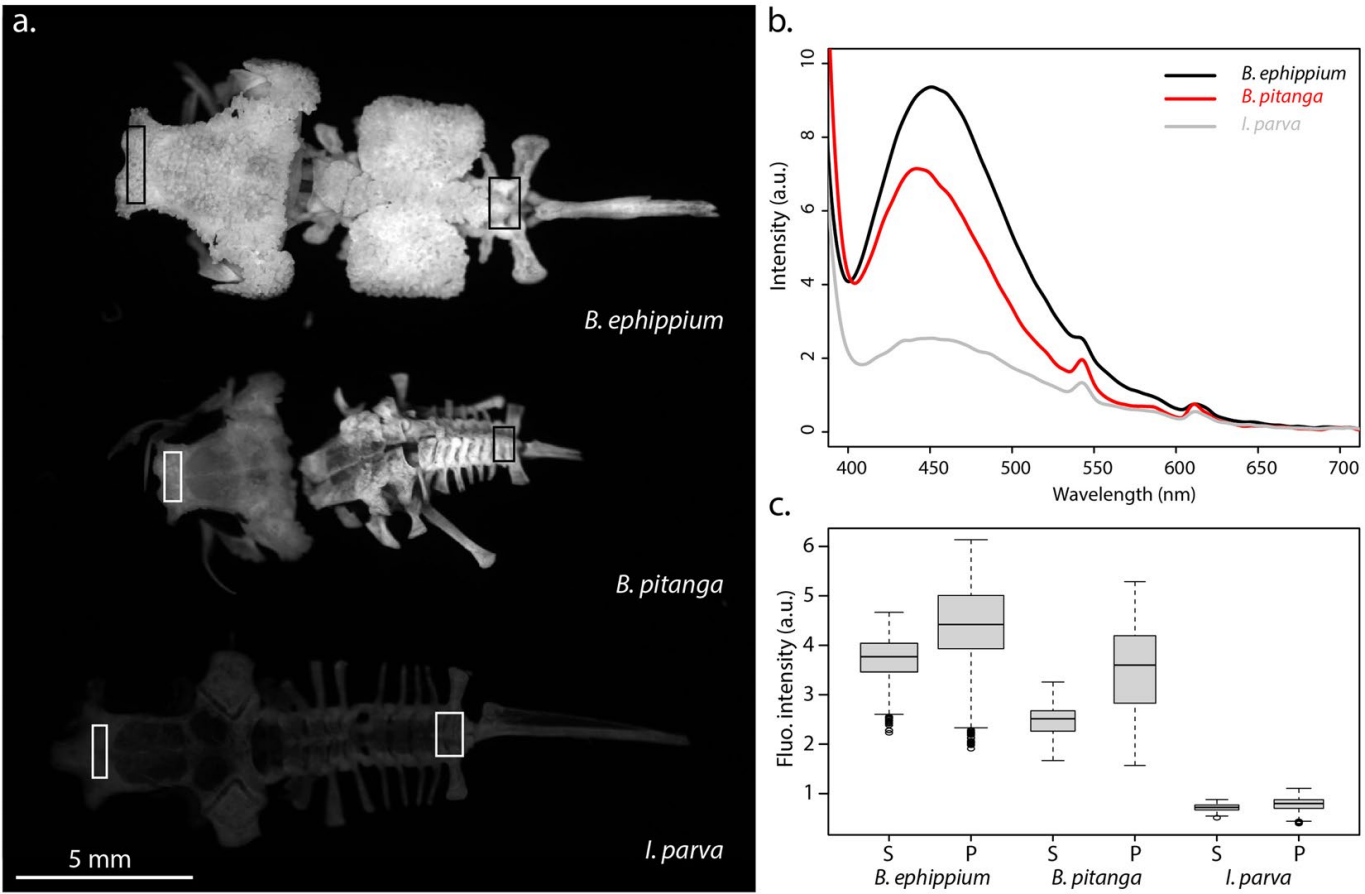
Bone fluorescence in pumpkin toadlets. (**a**) Emission of bones of *Brachycephalus ephippium*, *B*. *pitanga* and *Ischnocnema parva* under UVA lighting (λ_excitation_ = 365 nm) collected in the spectral range where their fluorescence occurs, using a 455–485 nm band-pass filter. The bones represented are the skull, spinal column and urostyle. All were imaged in a single shot to allow direct comparison of their emission intensities. (**b**) Emission spectra of the skull (1.5 mm diameter area) of the three species under UV lighting (λ_excitation_ = 365 nm), in arbitrary units (a.u.). (**c**) Boxplots, corresponding to the boxes in (**a**), comparing fluorescence intensities (in terms of grey-level of single pixels) of the skull (S) and pelvic area (P) of one individual for each of the three species (*B*. *ephippium*: n = 1,760 pixels (S), n = 2,331 pixels (P); *B*. *pitanga*: n = 1,064 pixels (S), n = 943 pixels (P); *I*. *parva*: n = 1,054 pixels (S), n = 1,320 pixels (P)). The boxes represent the first and the third quartiles; the bold line the median; the whiskers the first and ninth deciles; and the points outliers. Quantitative indices are given in Table S1.

**Figure 5:**
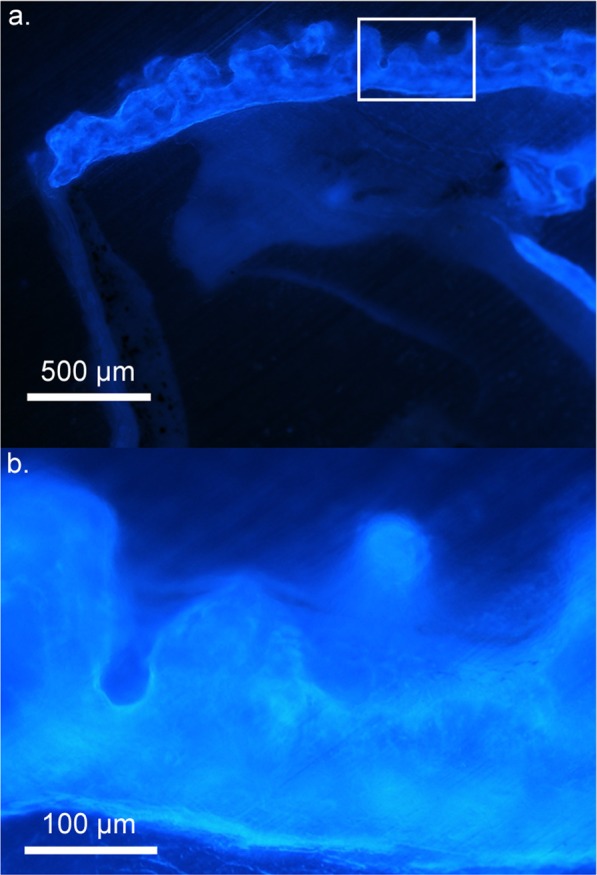
Fluorescence distribution within the bone in *Brachycephalus ephippium*. Photomicrograph of a transverse, non-decalcified section of the dorsal bony plates at 10x (**a**) and an enlargement of the boxed area at 40x magnification (**b**). The section is illuminated with UV-A light (λ_excitation_ = 365 nm) and no emission filter was used.

This unique fluorescence was further characterized by illuminating the pumpkin toadlets (n = 2 *B*. *ephippium*; n = 2 *B*. *pitanga*) only with UV light (Fig. 1c,g), which eliminates visible light emissions other than UV-excited fluorescence (for example, it avoids reflectance of purple light emitted by commercially-available UV torches, a phenomenon generating the apparently different colouration of the different toadlets in Fig. 1b,f,j). Bone fluorescence was maximal when the frogs were illuminated with 365–385 nm (UVA) light (Fig. S2). The examination of prepared skeletons under such conditions (using LED with λ_excitation_ centred at 365 nm and a 30 nm bandwidth) reveals that fluorescence is not restricted to the bony tubercles on the head and back of pumpkin toadlets, but occurs throughout the entire skeleton (Fig. 4a). UV emission spectra collected from the skull of *Brachycephalus* species and *Ischnocnema parva*, a closely related, non-fluorescent species (Fig. 1i–k), show that bone emits fluorescence centred at 450–470 nm, albeit at a much lower intensity in *I*. *parva* (Fig. 4b). The ossified tissue of *B*. *ephippium* and *B*. *pitanga* is highly fluorescent compared to that of *I*. *parva*: 5.18 and 3.43 times higher for the skull, respectively, and 5.65 and 4.45 times for the pelvic region, respectively (Fig. 4a,c). Within *Brachycephalus* spp., bone fluorescence intensity also varied, with the bones of *B*. *ephippium* being more fluorescent than those of *B*. *pitanga*:1.5 and 1.27 times higher for the skull and pelvic region, respectively (Fig. 4c). Moreover, fluorescence is not homogeneous within the bony structures: peripheral portions of the bone fluoresce more than cortical bone (Fig. 5).

## Discussion

### Source of fluorescence in pumpkin toadlets

We have discovered that striking fluorescent patterns are visible in life in two species of pumpkin toadlets (*B*. *ephippium* and *B*. *pitanga*), and that they are generated by dermal ossifications. This fluorescence is different from that described in the South American treefrogs *Boana punctata* and *B*. *atlantica* (Hylidae), the skin of which is fluorescent under 390–430 nm (purple-dark blue) light, and does not form any pattern^3^.

Fluorescent patterns created by bone have recently been described in chameleons^4^. Bones are naturally fluorescent^18–20^, but bone fluorescence is normally very weak and not visible through the tissues of living animals. As in *B*. *ephippium* and *B*. *pitanga*, bone fluorescence in chameleons is visible due to the thinness of the epidermal layer overlying bony tubercles, which allows UV light to penetrate and the fluorescence to be emitted back through the skin. Prötzel and colleagues (2018) did not quantify or compare the fluorescence of the visible tubercles to the rest of the specimens’ skeletons or to the bones of other chameleons. Thus, it remains unknown whether the examined species exhibit a unusually high level of fluorescence compared to other chameleons.

Chemically, bone fluorescence in living animals is largely attributed to collagen^18,19^, and particularly to the aromatic amino acids within it (tryptophan, phenylalanine and tyrosine). The peak emission wavelength of around 470 nm of pumpkin toadlets’ ossified tissues under UV lighting (λ_excitation_ = 365 nm) is consistent with spectra of whole bone, type I collagen and apatite described by Bachman and Ellis^18^. However, this spectral characterization is not sufficiently precise to exclude other compounds that could contribute to fluorescence at the same peak emission wavelength. The fact that bones of *B*. *ephippium* and *B*. *pitanga* are more fluorescent than those of *I*. *parva* may be the result of a greater collagen content in the bone, a different collagen composition/organisation, or additional compounds integrated in the bones of the fluorescent pumpkin toadlets. Structural chemical characterisation is needed to identify the fluorescent compounds, but this is beyond the scope of this paper.

### Potential function of fluorescence in pumpkin toadlets

Some fluorescent colours, which are not apparent to the human eye under natural lighting, are thought to be used by other animals as visual signals^1,5,8^. Pumpkin toadlets emit an unusual high-yield fluorescence intensity at 470 nm (blue) under UV illumination, raising the question of a potentially similar signalling function.

*Brachycephalus ephippium* and *B*. *pitanga* were recently shown to be deaf to their own advertisement calls^21^ and are known to use visual communication signals involving hand-waving and mouth-gaping^21,22^. In contrast, *B*. *hermogenesi*, a brown-coloured species which possesses a fully-developed inner ear^21^ and calls while hidden in the leaf litter^23^, lacks dermal ossification, which is only found in lighter-coloured *Brachycephalus* species including *B*. *ephippium* and *B*. *pitanga*^24^. Light skin colour (relating to a lack of melanophores), fluorescent ossified tissue and loss of high-frequency hearing may therefore be linked in pumpkin toadlets. Furthermore, the morphology of the ossified dermal plates appearing under UV illumination in pumpkin toadlets is species-specific^24^, and in *B*. *ephippium* these plates are fully developed only in sexually mature individuals (see Figs 2 and S1). Fluorescence could be used to enhance intraspecific visual communication, as shown in certain shrimp, spiders and birds^1,2,8^. The fact that the fluorescent patterns are on the dorsal surfaces of these frogs does not rule out a potential role in intraspecific signalling, given that these animals inhabit a three-dimensional forest environment in which individual frogs will often have vantage points allowing them to see the backs of others. However, fluorescent patterns could alternatively reinforce aposematic coloration in the toxic pumpkin toadlets^25^, used as a warning signal directed at potential predators such as spiders or birds, which are known to detect fluorescent patterns under natural light in other behavioural contexts^1,2^.

Although it is tempting to suggest that fluorescence in *Brachycephalus* has a visual signalling function, it is important to note that this inference rests on a pattern identification made using intense, artificial UV sources. In natural habitats, UV intensity is much lower, and reflectance of visible wavelengths is not dissociable from UV-based fluorescence. In an attempt to quantify the respective contributions of reflectance and fluorescence, we subjected the most fluorescent pumpkin toadlet, *B*. *ephippium*, to various combinations of UVA and ‘pseudo-natural’ lighting (i.e. broad-spectrum illumination with realistic UV content, reproduced in the lab; see SI text and Fig. S3A–C). Under pseudo-natural conditions without elevated UV, fluorescence emission appeared 43.09 times lower in intensity than reflectance, i.e., fluorescence only contributed 2.32% of the total light emitted by the frog (Fig. S3A–C,F). In other words, fluorescence under these conditions is overwhelmed by reflectance, and the human eye sees only the reflected orange colour of these frogs. Only with the addition of very intense, artificial UV does the fluorescent contribution become significant, and visible to the human eye.

Light spectral content varies significantly depending on the environment, the weather and the time of the day^26^. *Brachycephalus ephippium* and *B*. *pitanga* are diurnal anurans which live in leaf litter in Brazil’s Atlantic forest^14^. In the “sunny woodland shade” conditions assessed by Endler^26^, likely corresponding to the pumpkin toadlets’ natural habitat lightning conditions, short wavelength light (λ = 400 nm) is equal to or greater than the contribution at 470 nm, pumpkin toadlets’ fluorescence peak emission wavelength. Under such conditions, UV and short wavelength components of natural daylight might significantly excite bone fluorescence (Fig. S2). The vision of *Brachycephalus* species has not been tested, but other frog species are very sensitive to blue and green light^27,28^ and are much less sensitive to yellow, orange and red, the skin colorations of the two species studied. Even so, the low ratio of fluorescence to reflectance which we have documented here means that the eyes of *Brachycephalus*, or any other species, would have to be particularly sensitive in the blue-green range in order to distinguish the fluorescence effect of the dermal bone from the background. Whether any species possesses such sensitivity, and can thus make use of the cues available from dermal bone fluorescence, remains to be established.

The present study adds to the growing list of documented cases of fluorescence in terrestrial animals. Bone fluorescence within the *Brachycephalus* genus appears to be associated with the loss of high-frequency hearing, raising the possibility that it represents an alternate communication channel. However, data are not available from enough species to be sure that this correlation is robust, and the functions of these fluorescent patterns remain speculative. Thorough *in situ* light spectra measurements and behavioural studies are needed in order to determine whether conspecifics and/or potential predators respond to fluorescent patterns in *Brachycephalus* toadlets, and in what way. The biochemical nature of this fluorescence remains to be investigated.

## Methods

### Animals

Animals were visually and acoustically located and collected at the Parque Estadual da Serra do Mar, state of São Paulo, Brazil between February and April 2016 (IBAMA collecting permit, 27745-9; COTEC state permit: 468/2015 D028/2015). Animals were classified as adult males when they were collected while calling, or as adult females if they had mature oocytes in their ovaries, visible through the ventral skin. Some individuals were dissected after the experiments to verify the presence of vocal slits (found only in sexually-mature males) and the development of their reproductive organs. Animals were kept in terraria, each containing up to ten individuals, at 23 °C, with natural light from 06:00 to 18:00. A high humidity level was maintained by misting the terraria every other day. Larger individuals were fed fruit flies (*Drosophila melanogaster*); smaller ones fed on micro-invertebrates such as springtails (*Collembola* spp.) present in the leaf litter, which was changed regularly. Animals were euthanized by cutaneous application of 20% benzocaine gel. No experiment was performed on live animals and euthanasia was performed in accordance with relevant guidelines and regulations. Originally, our study aimed at understanding hearing in these frogs, and we obtained ethical approval of our experiments and animal keeping from the College of Science Research Ethics Committee (Ethics permit CORSEC. 111) and the Danish National Animal Experimentation board (permit N°2015-15-0201-00619^21^). We discovered the fluorescence of the frogs during this research on hearing, and used the same individuals. Only photos were taken in addition to the hearing experiments that were approved by the ethical committees, and all subsequent experiments were performed on fixed specimens. Specimens from the collection of the Institute Butantan, São Paulo, Brazil, were used for the imaging and spectral analyses (loan #23112016).

### Fluorescence photography

Fluorescent colour patterns of *B*. *ephippium* and *B*. *pitanga* were revealed using three different experimental set-ups. A Nikon D800 camera was used to photograph specimens (n = 3 *Brachycephalus ephippium*; n = 1 *Brachycephalus pitanga*) illuminated with two Fluotest Forte UV (λ_excitation_ centred around 365 nm) quartz lamps emitting at 180 W (Fig. 1b,f,j). A Pentax 645Z camera was used to photograph specimens (n = 10 *B*. *ephippium*) illuminated with two SANKYO UV lamps (λ_excitation_ = 315–400 nm) emitting at 8 W (Figs 2 and S1) and a Panasonic DMC-ZS40 camera was used to photograph specimens (n = 2 *B*. *ephippium*) illuminated with a single LED INOVA UV microlight (λ_excitation_ = 365–400 nm; Video S1).

### Micro-computed tomography

Ethanol-preserved specimens of *Brachycephalus ephippium* (n = 1), *B*. *pitanga* (n = 1) and *Ischnocnema parva* (n = 1) were wrapped in cellophane to minimize drying during the scans, which were made using a Nikon XT H 225 µCT scanner. The settings used were 130 kV and 110 µA. Images were constructed from 1080 projections, each with 1000 ms exposure and two frames averaged per projection. The scan data were processed with CT Agent XT 3.1.9 and CT Pro 3D XT 3.1.9 (Nikon Metrology, 2004–2013). Cubic voxel side-lengths were 14.5 μm. TIFF stacks from the scans were converted to JPEG in Adobe Photoshop CS 8.0 (Adobe Systems Inc., 2003) and then reconstructed using MicroView 2.5.0 (Parallax Innovations, Ilderton, Canada).

### Histology

Specimens (n = 3 *B*. *ephippium*; n = 1 *B*. *hermogenesi*; n = 4 *B*. *pitanga*) were fixed in 4% formaldehyde in phosphate-buffered saline or Karnovsky solution for 48 hours. They were subsequently transferred to 70% ethanol. After two to eight weeks of decalcification in a constantly spinning bath of 4% ethylenediaminetetraacetic acid (EDTA), specimens were gradually dehydrated using up to 100% ethanol and embedded in glycol methacrylate (Leica Historesin). Serial sections (4 µm thickness) were made with a microtome (Leica RM2255) using glass knifes. Histological sections were stained with toluidine blue and fuchsine to reveal cellular structures. Photomicrographs were obtained using an Olympus BX51 microscope equipped with a digital camera and Image-Pro Express software, version 5.0 (Media Cybernetics).

### Whole skeleton preparation

Alcohol-preserved specimens of *B*. *ephippium* (n = 2), *B*. *pitanga* (n = 2) and *I*. *parva* (n = 1) were immersed in diluted bleach solution for 30 s and rinsed in a distilled water bath in order to remove the flesh from the bones. The operation was reiterated if a significant amount of flesh was remaining. Specimens were closely monitored to ensure that bleach did not damage the bones. Any remaining flesh was gently scraped from the bones with a toothpick.

### Non-decalcified serial sections

Specimens were fixed in 70% ethanol and gradually dehydrated using up to 100% ethanol. Full specimens were then embedded in methyl methacrylate resin and processed for non-decalcified histology. Each transversal bone section was cut into 100 µm-thick sections using a circular, water-cooled, diamond saw (Leitz 1600; Leica Biosystems, Nussloch, Germany).

### Multispectral imaging, quantification of bone luminescence and spectroscopy

The distribution and relative intensity of the reflectance and fluorescence signals emitted from different excitation lights were assessed using multispectral imaging, at the macroscale on entire specimens and on the prepared skeletons. The multispectral setup consists of a low-noise 1 megapixel Si EM_CCD camera (Qimaging Rolera EM-C^2^) fitted with a UV-VIS-IR 60 mm 1:4 Apo Macro (CoastalOptics). Spectral interference band-pass filters (Andover Corp) were used to collect images in specific spectral ranges. Illumination was provided by 16 LED lights, ranging from the UV-A (365–385 nm) to the near infrared (~800 nm) (CoolLED pE-4000), coupled to a liquid light-guide fiber fitted with a fiber-optic ring light-guide, allowing homogeneous illumination. Combinations of wavelengths were used to reproduce natural lighting conditions.

Bone fluorescence of *B*. *ephippium*, *B*. *pitanga* and *I*. *parva* (n = 1 individual for each species) under UVA (365–385 nm) was compared by recording all three skeletons in a single image, in the spectral range where fluorescence occurs, i.e. around 470 nm, using a 455–485 nm band-pass filter. The relative contributions of fluorescence vs. reflectance in the fluorescing bony tubercles and inter-tubercles spaces of *B*. *ephippium* was assessed by comparing emission intensities in the same spectral range, while changing lighting conditions. In parallel, emission spectra were collected on the dorsal bony plate of *B*. *ephippium* using a JETI SPECBOS 1211UV spectrometer (see Supplementary Material).

Emission intensities were extracted using ImageJ^29^. Pixel attribution to tubercles and inter-tubercles spaces was made on perfectly registered images using a mask manually drawn from the image collected under pure UVA illumination. Statistical tests were performed using the R statistical environment. Outliers were eliminated using three-sigma.

## Supplementary information

Supplementary information for: “Extraordinary bone fluorescence reveals hidden patterns in pumpkin toadlets”

Video S1. Fluorescence in live adult Brachycephalus ephippium.

## Data Availability

All data produced in this study are included in the text and supplementary information documents.

## References

[1] Arnold KE, Owens IPF, Marshall NJ (2002). Fluorescent signaling in parrots. Science.

[2] Lim MLM, Land MF, Li D (2007). Sex-specific UV and fluorescence signals in jumping spiders. Science.

[3] Taboada, C. *et al*. Naturally occurring fluorescence in frogs. *Proc*. *Natl*. *Acad*. *Sci*. 201701053, 10.1073/pnas.1701053114 (2017). 10.1073/pnas.1701053114 PMC5389305 28289227

[4] Prötzel D (2018). Widespread bone-based fluorescence in chameleons. Sci. Rep..

[5] Gruber DF (2016). Biofluorescence in catsharks (Scyliorhinidae): fundamental description and relevance for elasmobranch visual ecology. Sci. Rep..

[6] Sparks JS (2014). The covert world of fish biofluorescence: a phylogenetically widespread and phenotypically variable phenomenon. Plos One.

[7] Finkbeiner SD, Fishman DA, Osorio D, Briscoe AD (2017). Ultraviolet and yellow reflectance but not fluorescence is important for visual discrimination of conspecifics by *Heliconius erato*. J. Exp. Biol..

[8] Mazel CH, Cronin TW, Caldwell RL, Marshall NJ (2004). Fluorescent enhancement of signaling in a mantis shrimp. Science.

[9] Gaffin DD, Bumm LA, Taylor MS, Popokina NV, Mann S (2012). Scorpion fluorescence and reaction to light. Anim. Behav..

[10] Gruber DF (2015). Adaptive evolution of eel fluorescent proteins from fatty acid binding proteins produces bright fluorescence in the marine environment. Plos One.

[11] Davis MP, Sparks JS, Smith WL (2016). Repeated and widespread evolution of bioluminescence in marine fishes. Plos One.

[12] Gruber, D. F. & Sparks, J. S. First observation of fluorescence in marine turtles. *Am*. *Mus*. *Novit*. 1–8, 10.1206/3845.1 (2015).

[13] Hadjioloff AI, Zvetkova E (1970). Contribution à l’étude de la fluorescence propre de la peau des amphibiens (*Bombina variegata* et *Rana esculenta*). Comptes Rendus Assoc. Anat..

[14] Haddad, C F. B. *et al*. *Guide to the amphibians of the atlantic forest: diversity and biology*. (Anolis Books, 2013).

[15] Bagnara, J. T. Pigment cells. in *Biology of the integument* 136–149, 10.1007/978-3-662-00989-5_8 (Springer, Berlin, Heidelberg, 1986).

[16] Kollias N, Sayre RM, Zeise L, Chedekel MR (1991). New trends in photobiology: photoprotection by melanin. J. Photochem. Photobiol. B.

[17] Hofer R, Mokri C (2000). Photoprotection in tadpoles of the common frog, *Rana temporaria*. J. Photochem. Photobiol. B.

[18] Bachman CH, Ellis EH (1965). Fluorescence of bone. Nature.

[19] Ren P-G, Ma T, Huang Z, Smith RL, Goodman SB (2008). Quantitation of bone area in undecalcified frozen sections with fluorescent microscopy. J. Histotechnol..

[20] Swaraldahab MAH, Christensen AM (2016). The effect of time on bone fluorescence: implications for using alternate light sources to search for skeletal remains. J. Forensic Sci..

[21] Goutte S (2017). Evidence of auditory insensitivity to vocalization frequencies in two frogs. Sci. Rep..

[22] Pombal J, Sazima I, Haddad C (1994). Breeding behavior of the pumpkin toadlet, *Brachycephalus ephippium* (Brachycephalidae). J. Herpetol..

[23] Verdade VK (2008). Advertisement call, vocal activity, and geographic distribution of *Brachycephalus hermogenesi* (Giaretta and Sawaya, 1998) (Anura, Brachycephalidae). J. Herpetol..

[24] Clemente-Carvalho RBG (2009). Hyperossification in miniaturized toadlets of the genus *Brachycephalus* (Amphibia: Anura: Brachycephalidae): microscopic structure and macroscopic patterns of variation. J. Morphol..

[25] Pires OR (2002). Occurrence of tetrodotoxin and its analogues in the Brazilian frog *Brachycephalus ephippium* (Anura: Brachycephalidae). Toxicon.

[26] Endler JA (1993). The color of light in forests and its implications. Ecol. Monogr..

[27] Govardovskiĭ VI, Zueva LV (1974). Spectral sensitivity of the frog eye in the ultraviolet and visible region. Vision Res..

[28] Yovanovich CAM (2017). The dual rod system of amphibians supports colour discrimination at the absolute visual threshold. Phil Trans R Soc B.

[29] Schneider CA, Rasband WS, Eliceiri KW (2012). NIH Image to ImageJ: 25 years of image analysis. Nat. Methods.

