# Supplementary material for: Intense bone fluorescence reveals hidden patterns in pumpkin toadlets
Source: Sci Rep. 2019 Mar 29;9:5388. doi: 10.1038/s41598-019-41959-8 (PMC6441030; doi:10.1038/s41598-019-41959-8)
Supplement: Supplementary file 1 — Supplementary information for: “Extraordinary bone fluorescence reveals hidden patterns in pumpkin toadlets” [file 41598_2019_41959_MOESM1_ESM.pdf]

Supplementary information for: "Extraordinary bone fluorescence reveals hidden patterns in pumpkin toadlets"

Sandra Goutte*, Matthew J. Mason, Marta M. Antoniazzi, Carlos Jared, Didier Merle, Lilian Cazes, Luís Felipe Toledo, Hanane el-Hafci, Stéphane Pallu, Hugues Portier, Stefan Schramm, Pierre Gueriau, Mathieu Thoury

Table S1. Bone fluorescence quantification in pumpkin toadlets. Minimal, maximal and mean (± standard deviation) pixel grey-level intensities (16-bits) corresponding to the boxplots shown in Fig. 4a after elimination of the outliers (mean ± 3-sigma). For each dataset, the number of pixels (including outliers) is given (n).

	min	max	mean ± sd	n
B. ephippium skull	25108	46672	37325.87 ± 3983.96	1760
B. ephippium urostyle	20072	61360	44201.60 ± 7992.32	2331
B. pitanga skull	16688	32560	24663.85 ± 3213.28	1064
B. pitanga urostyle	15684	52880	34829.80 ± 9422.83	943
I. parva skull	5456	8796	7189.59 ± 640.96	1054
I. parva urostyle	3940	11056	7823.56 ± 1375.69	1320

Video S1. Fluorescence in live adult *Brachycephalus ephippium*. Alternate natural and UV lightings show fluorescent patterns in a walking pumpkin toadlet. A single LED INOVA UV microlight (365-400 nm) and a Panasonic DMC-ZS40 camera were used.

Figure S1. 3D Fluorescence spectra of the bone of *Brachycephalus ephippium.*

The steady-state fluorescence of *B. ephippium* bone (dorsal plate) was measured using a Jasco FP-8500 Fluorescence Spectrometer (Jasco GmbH, Groß-Umstadt, Germany) with 5 nm excitation and emission bandwidths using a ILFC-847 liquid N2 coolable 100 mm integrating sphere. The empty diagonal area represents non-collected values for $\lambda_{emission} = \lambda_{excitation}$, which would correspond to reflectance and not fluorescence. Pumpkin toadlets' bone fluorescence is excited by long wavelength UV as well as short wavelength visible light (320 – 450 nm).

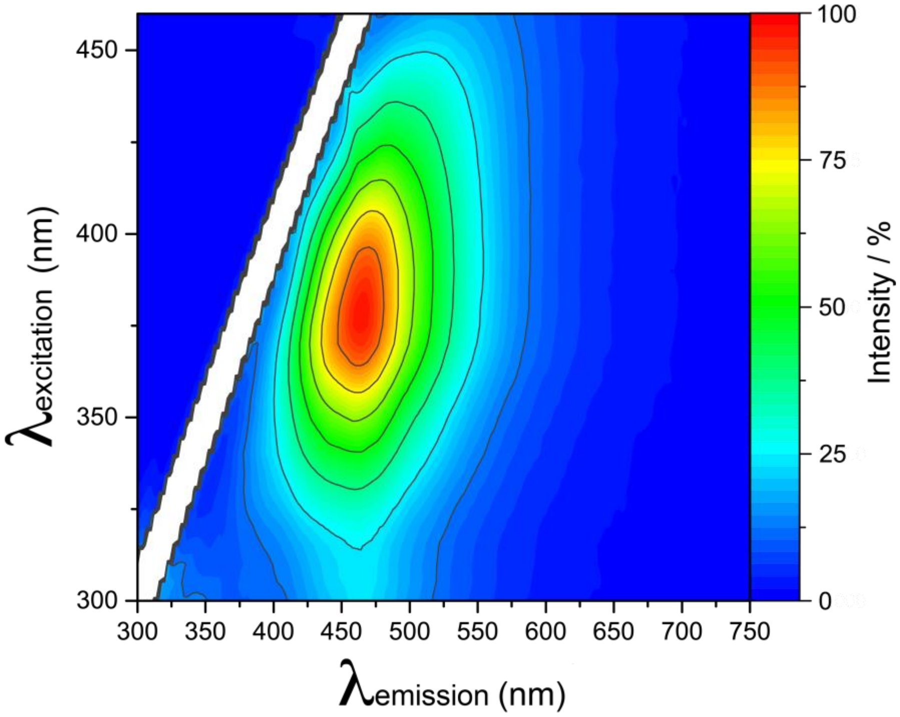

Figure S2. Quantification of fluorescence vs. reflectance contributions in an ethanol-preserved specimen of *Brachycephalus ephippium*. (A–D) Emission spectra from the dorsal bony plates (the exact area is shown by the red circles in A'–D') subjected to (A) intense UVA (λ=385±10 nm;(B) pseudo-natural light with the same (unrealistically high) UVA content; (C) pseudo-natural light with realistic UVA content and (D) light with λ=470±20 nm (the domain where the pumpkin toadlets emit their fluorescence. In grey, the spectral radiance of the source collected from reflectance measurements on a Spectralon white reference. In black, the reflectance (and luminescence) signal collected from the dorsal bony plates. As only the bony tubercles (t.) emit fluorescence, we also represented separately the part of the emission spectrum due to the bony tubercles and to the inter-tubercle (inter-t.) spaces (B; see also SI text). (A'–D') Emission of the entire specimen collected in the spectral range where fluorescence occurs using a 455-485 nm band-pass filter (highlighted by the light grey boxes in A–D) under A–D lighting conditions, respectively. All images were collected using the same exposure time, and all but A' (much less intense signal) are shown using the same grey scale for comparison; grey scale for A' has been increased 70 times to ease visualization. (A''–D'') Close up on the area on which spectroscopy measurements were collected. (E) Mask attributing pixels to the fluorescent bony t. and inter-t. spaces. (F) Boxplots showing grey-level intensities in the bony t. and inter-t. spaces for the different lighting conditions. Differences between the two are indicated, as well as contribution of fluorescence vs. reflectance in the bony t. under realistic and unrealistic pseudo-natural lighting. Scale bar in A'–D' represents 5 mm.

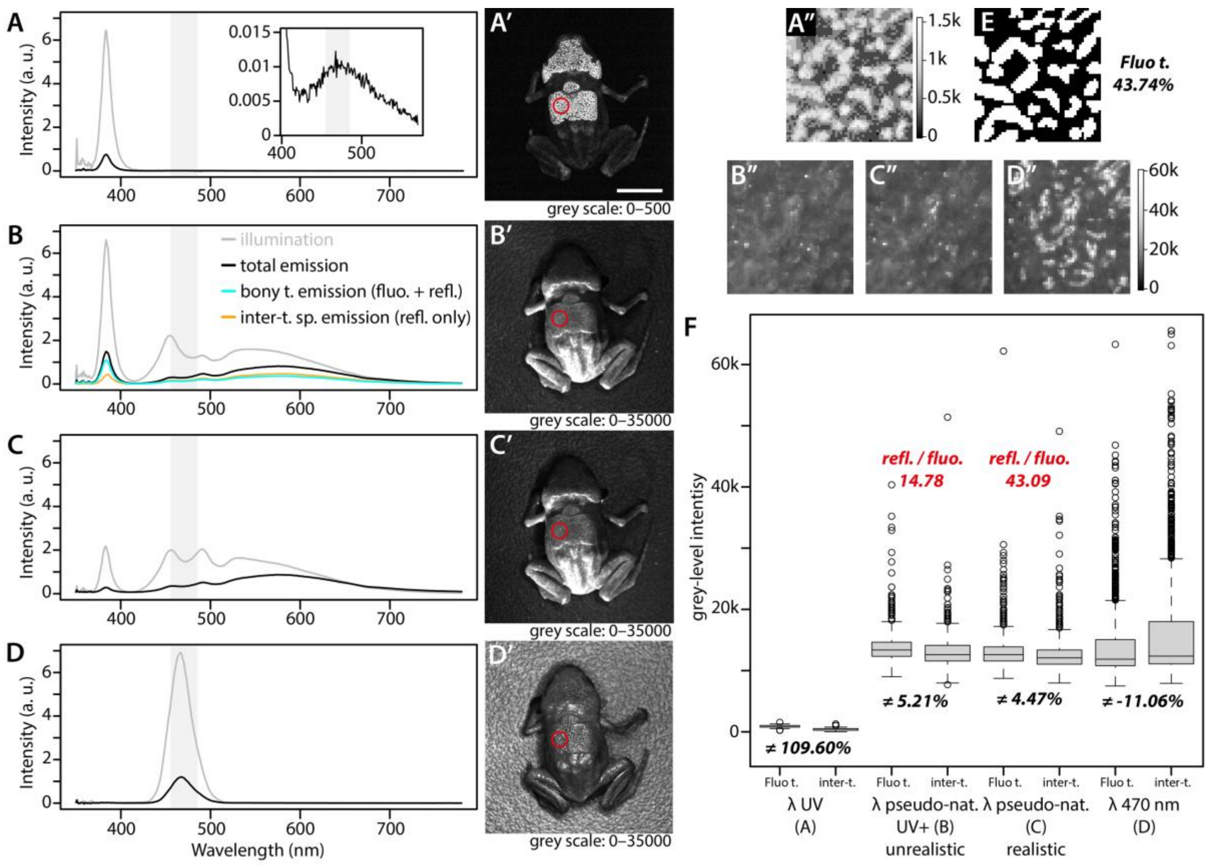
A
Intensity (a. u.)
0.015
0.01
0.005
400
500
600
700
A′
grey scale: 0–500
A″
1.5k
1k
0.5k
0
E
Fluo t.
43.74%
B
illumination
total emission
bony t. emission (fluo. + refl.)
inter-t. sp. emission (refl. only)
B′
grey scale: 0–35000
B″
C″
D″
60k
40k
20k
C
C′
grey scale: 0–35000
D
D′
grey scale: 0–35000
Wavelength (nm)
F
grey-level intentisy
refl. / fluo.
14.78
refl. / fluo.
43.09
≠ 109.60%
≠ 5.21%
≠ 4.47%
≠ -11.06%
Fluo t.
inter-t.
λ UV
(A)
λ pseudo-nat.
UV+ (B)
unrealistic
λ pseudo-nat.
(C)
realistic
λ 470 nm
(D)

Figure S3. **Dorsal plate morphology and sexual maturity**. Live sub-adult (left) and adult (right) *Brachycephalus ephippium* photographed under regular lighting (a) and 365nm UV lighting (b). The two individuals have the same snout-vent length (sub-adult: 15.0 mm, adult: 14.9 mm) but the dorsal plates are not fully developed in the sub-adult, which is only visible under UV lighting.

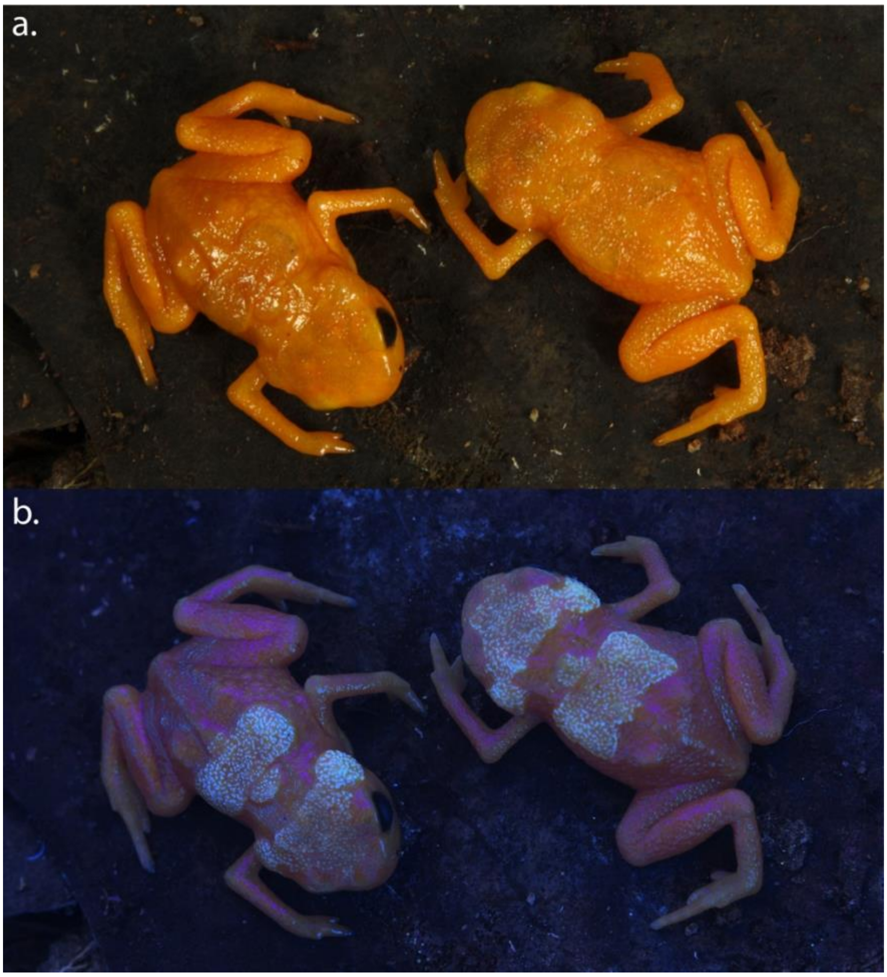

Supporting text

Quantification of fluorescence vs. reflectance contributions in pumpkin toadlets

In the main text of the article we briefly discuss the important issue that, in natural lighting conditions, fluorescence would only represent a minute portion of the total light emitted by the frog, and as such would likely be overwhelmed by reflectance of visible light. We have addressed this question by quantifying, in the laboratory, the respective contribution of fluorescence vs. reflectance in the most fluorescent pumpkin toadlet, *Brachycephalus ephippium*. In order to reproduce 'realistic' pseudo-natural light, we coupled a laboratory lamp with narrow-wavelength LED lights of the CoolLED pE-4000 universal LED illumination system, allowing us *almost* to homogenize illumination over the whole UVA and visible spectral range. By changing intensities of the different LEDs, it was possible to simulate different daylight conditions (Figure S2B, C).

We collected emission spectra from the dorsal bony plates of an ethanol-preserved specimen of *B. ephippium* under the different illumination created: intense UVA (λ=385±10 nm), pseudo-natural light with the same (unrealistic) intense UVA content, pseudo-natural light with realistic UVA content, and dark blue light (centred at 470 nm, the domain where the pumpkin toadlets emit their fluorescence) (Figure S2A–D). Under both pseudo-natural illumination conditions, fluorescence appears weak compared to reflectance in the pumpkin toadlets (Figure S2B-C). Note that although the frog is orange and mostly reflects light in the orange spectral range, it also reflects (albeit at 2–3 times lower intensity) other visible wavelengths such as dark blue in regions where the bony fluorescence occurs (Figure S2B–C).

We also collected emission distributions over the entire specimen under the different lighting conditions in the spectral range where fluorescence occurs, using a 455-485 nm band-pass filter (Figure S2A'–D'). By using this filter, we simulated the most favourable conditions for a potential visual function of fluorescence, as it selectively considers the fluorescent signal while rejecting the orange reflectance of the frog skin. Under our pseudo-natural conditions, even with unrealistically high UVA content, the pattern generated by the tubercles of the dermal ossified plates is hardy distinguishable (Figure 1 and S2B'–C'). If we zoom in on the area studied by spectroscopy, the pattern is barely visible (Figure S2B''–C''). A potential function in visual signalling would therefore require the eye of the pumpkin toadlets (or other animals) to have a high sensitivity.

As the images under UVA and pseudo-natural conditions were collected in a way that grey-level intensity can directly be compared, we extracted mean intensities from the pixels corresponding to the fluorescent bony tubercles and to the inter-tubercle spaces (Figure S2F), using a mask manually drawn from the image collected under pure UVA illumination (Figure S2E). Before extracting the grey-level intensities, the different images were registered with subpixel accuracy to allow comparisons. Outliers were eliminated using three-sigma. While average emission intensity from the bony tubercles was 109.6% higher than that of inter-tubercle spaces under UVA illumination only, it was only 4.47% higher under pseudo-natural conditions with realistically high UV content (5.21% under unrealistically intense UV content; Figure S2F).

From the mask, we assessed the contribution of fluorescence vs. reflectance. Fluorescence intensity in the bony tubercles is precisely known from the image collected under UVA only. Under the pseudo-natural conditions, reflectance intensity in these pixels can be

calculated by subtracting this fluorescence intensity from the total signal emitted (which corresponds to reflectance + fluorescence) . Fluorescence emission appears 43.09 times lower in intensity than reflectance, i.e. fluorescence only contributes 2.32% of the total signal under pseudo-natural conditions with realistically high UV content; and still contributes very little (14.78 times lower intensity, 6.77% contribution) under unrealistically intense UV content.
